# Kempopeptin C, a Novel Marine-Derived Serine Protease Inhibitor Targeting Invasive Breast Cancer

**DOI:** 10.3390/md15090290

**Published:** 2017-09-16

**Authors:** Fatma H. Al-Awadhi, Lilibeth A. Salvador, Brian K. Law, Valerie J. Paul, Hendrik Luesch

**Affiliations:** 1 Department of Medicinal Chemistry, College of Pharmacy, University of Florida, Gainesville, FL 32610, USA; fatalawadhi@ufl.edu; 2 Center for Natural Products, Drug Discovery and Development (CNPD3), University of Florida, Gainesville, FL 32610, USA; bklaw@ufl.edu; 3 Marine Science Institute, College of Science, University of the Philippines, Diliman, Quezon City 1100, Philippines; lsreyes@msi.upd.edu.ph; 4 Department of Pharmacology and Therapeutics, College of Medicine, University of Florida, Gainesville, FL 32610, USA; 5 Smithsonian Marine Station, 701 Seaway Drive, Fort Pierce, FL 34949, USA; paul@si.edu

**Keywords:** marine cyanobacteria, serine proteases, migration, breast cancer

## Abstract

Kempopeptin C, a novel chlorinated analogue of kempopeptin B, was discovered from a marine cyanobacterium collected from Kemp Channel in Florida. The structure was elucidated using NMR spectroscopy and mass spectrometry (MS). The presence of the basic Lys residue adjacent to the *N*-terminus of the 3-amino-6-hydroxy-2-piperidone (Ahp) moiety contributed to its selectivity towards trypsin and related proteases. The antiproteolytic activity of kempopeptin C was evaluated against trypsin, plasmin and matriptase and found to inhibit these enzymes with IC_50_ values of 0.19, 0.36 and 0.28 μM, respectively. Due to the significance of these proteases in cancer progression and metastasis, as well as their functional redundancy with respect to targeting overlapping substrates, we examined the effect of kempopeptin C on the downstream cellular substrates of matriptase: CDCP1 and desmoglein-2 (Dsg-2). Kempopeptin C was shown to inhibit the cleavage of both substrates in vitro. Additionally, kempopeptin C reduced the cleavage of CDCP1 in MDA-MB-231 cells up to 10 µM. The functional relevance of targeting matriptase and related proteases was investigated by assessing the effect of kempopeptin C on the migration of breast cancer cells. Kempopeptin C inhibited the migration of the invasive MDA-MB-231 cells by 37 and 60% at 10 and 20 µM, respectively.

## 1. Introduction

Cancer is a major public health problem and the second contributor to deaths in the United States. In 2017, it has been estimated that ~600,920 cancer-related deaths will occur in the United States [1]. Tumor metastasis, the dissemination of cancer cells from their primary site to distant organs, is considered a leading cause of death among cancer patients [2]. One of the earliest events involved in metastasis is the degradation of extracellular matrix (ECM) components and adhesive molecules, a process often carried out by proteases.

Among the different protease families, serine proteases have been reported to be aberrantly expressed and implicated in the progression, invasion and metastasis processes of several cancers [3,4]. Of particular interest in the field of cancer research, a group of S1 serine proteases termed type II transmembrane serine proteases (TTSP) have been identified. TTSP are capable of degrading several extracellular matrix (ECM) components and activating certain substrates involved in the development and progression of cancers [4]. One such protease is matriptase, a type II transmembrane serine protease, which was originally discovered to be an ECM-degrading enzyme in breast cancer [5] and was identified to contribute to cancer invasiveness through its actions on substrates such as cell adhesion molecules [6], ECM proteins [7,8] and growth factors [9,10]. Matriptase is identified to be overexpressed in a variety of other tumors of epithelial origin [4] including colon, kidney, liver, lung, prostate, ovarian and primary breast carcinomas [11,12,13], with expression levels correlating with tumor grades [13]. Desmoglein-2 and CUB-domain containing protein 1 (CDCP1) are biomarkers of invasive cancers and known substrates of matriptase [14,15]. Desmogleins are glycoproteins and members of the cadherin superfamily of proteins being localized at the cell membrane [16,17]. CDCP1 is an integral highly glycosylated protein present at the cell surface and known to act as a target not only for matriptase, but also for other proteases such as plasmin [18]. Matriptase acts by shedding the cell surface desmoglein-2 (Dsg-2) and cleaving the membrane-bound extracellular domain of CDCP1 [14,15]; hence resulting in the disruption of tissue cohesiveness and the production of the cleaved form of CDCP1 implicated in the pro-invasive activities of CDCP1. Given the significance of matriptase in cancer biology, several studies attempted the development of agents targeting this protease [19,20,21].

**Figure 1:**
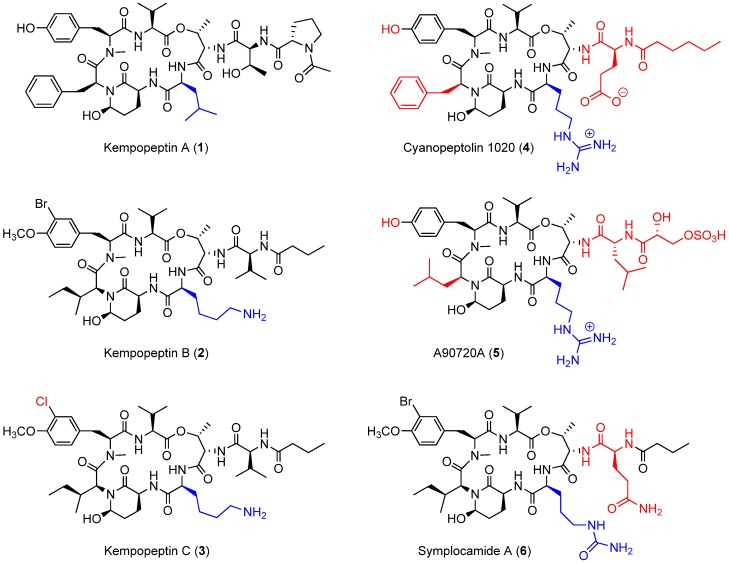
Kempopeptins A‒C (**1**‒**3**) and structurally-related 3-amino-6-hydroxy-2-piperidone (Ahp) containing cyclic depsipeptides bearing a basic residue (**4**‒**6**). The differences in the structures of **3**‒**6** compared to kempopeptin B (**2**) are highlighted.

Cyanobacterial secondary metabolites characterized by a 16-membered ring cyclic depsipeptide scaffold bearing the 3-amino-6-hydroxy-2-piperidone (Ahp) moiety are well recognized for their serine protease inhibitory activity against elastase, chymotrypsin and trypsin [22,23,24,25]. Lyngbyastatins 5‒10 and symplostatins 5‒10 are among the most potent marine-derived elastase inhibitors with IC_50_ values in the nanomolar range [22,23,25]. Previous studies have shown that the residue between the *N*-terminal end of Ahp and Thr binds to the enzyme’s specificity pocket and thus modulates the selectivity towards different serine proteases [26]. As an example, kempopeptins A (**1**) and B (**2**) (Figure 1), isolated from a Floridian marine cyanobacterium *Lyngbya* sp., exhibited serine protease inhibitory activity with different selectivity profiles [24]. Kempopeptin A, bearing the hydrophobic Leu residue, was found to exhibit selectivity towards elastase and chymotrypsin inhibition with IC_50_ values of 0.32 and 2.6 µM, respectively [24]. Kempopeptin B, on the other hand, bears a basic Lys residue and was found to exhibit selectivity towards inhibiting trypsin [24]. Here, we report the discovery and characterization of kempopeptin C (**3**), a novel chlorinated analogue of kempopeptin B, bearing a basic Lys residue adjacent to the Ahp moiety (Figure 1). We investigated its activity against the trypsin-related proteases plasmin and matriptase. Furthermore, we examined its effect on downstream cellular substrates of matriptase contributing to tumor invasiveness and metastasis.

## 2. Results and Discussion

### 2.1. Isolation and Structure Elucidation

The cyanobacterium collected from Kemp Channel in the Florida Keys was extracted and purified according to the procedure of Taori et al. (2008) [24], with directed purification of lyngbyastatin 7, kempopeptins and their analogues. This targeted approach afforded kempopeptin A (**1**) and the new compound kempopeptin C (**3**).

Kempopeptin C (**3**) gave an [M + Na]^+^ peak at *m/z* 971.5006 and an isotope peak at *m/z* 973.5002, with a 3:1 ratio, indicating the presence of one chlorine atom and a molecular formula of C_46_H_73_ClN_8_O_11_. The ^1^H NMR spectrum of kempopeptin C (**3**) displayed characteristic resonances for peptides and modified peptides corresponding to alpha protons (*δ*_H_ 4.28–4.45, 4.64–4.66, 5.05), *N*-CH_3_ protons (*δ*_H_ 2.74) and secondary amide protons (*δ*_H_ 7.35, 7.69, 7.83, 8.45) (Table 1 and Supporting Information). COSY, HSQC and HMBC analysis indicated that kempopeptin C (**3**) belonged to the Ahp-bearing family of cyclodepsipeptides and indicated the presence of the 2 × Val, Ile, Thr, Lys, *N*,*O*-diMe-Cl-Tyr and butyric acid. HMBC correlations established the connectivity and indicated that kempopeptin C (**3**) is the chloro analogue of kempopeptin B (**2**). This is supported by a mass difference of 43.9499 Da and the observed isotopic pattern.

The absolute configuration was determined by comparing the optical activity and ^1^H NMR spectra of kempopeptins B and C (**2** and **3**) (Supporting Information). The absolute configuration for kempopeptin B (**2**) was previously established [24] through acid hydrolysis and modified Marfey’s analysis, which indicated an L configuration of all amino acid residues, while the absolute configuration of the Ahp unit was established following CrO_3_ oxidation [24]. Analysis of ROESY spectra and proton-proton coupling constants of **3**, in comparison with kempopeptin B (**2**), established the relative configuration of the Ahp unit to be 3*S*, 6*R*, the same as kempopeptin B (**2**). The ^1^H NMR spectra for **2** and **3** (Supporting Information) were virtually identical, indicating the same relative configuration of the entire molecule. Comparison of the optical activity of **2** and **3** (−17.1 and −37.3, respectively) indicated that the configuration of both compounds (2 and 3) is the same.

### 2.2. Enzyme Inhibition Assays

**Figure 2:**
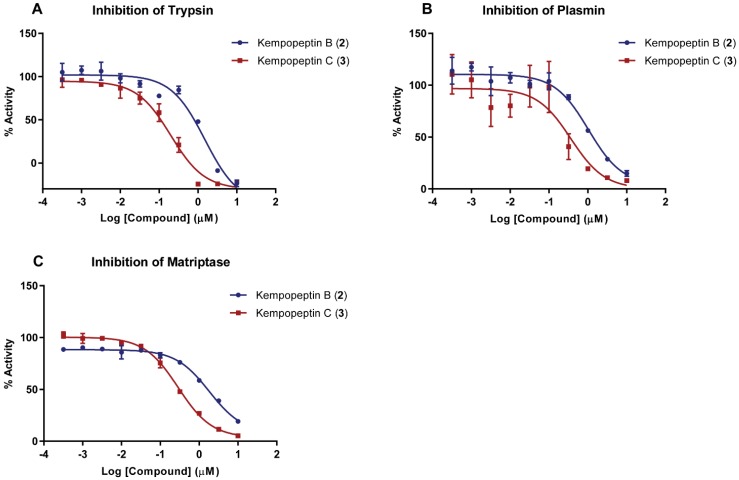
Dose-response curves for kempopeptins B and C (**2** and **3**) against serine proteases. (**A**) Trypsin; (**B**) plasmin; and (**C**) matriptase. The dose-response is presented as % fold inhibition against solvent control (DMSO). Data are presented as the mean ± SD, *n* = 3.

The presence of the modified amino acid residue Ahp and the basic Lys unit adjacent to its *N*-terminal end within the macrocycle of kempopeptins B and C (**2** and **3**) suggests potential serine protease inhibitory activity with selectivity towards trypsin and related enzymes with trypsin-like substrate specificities [24]. We therefore investigated the antiproteolytic activity of **2** and **3** against trypsin, plasmin and matriptase (Figure 2). Kempopeptin B (**2**) inhibited the enzymes tested with IC_50_ values of ~1 µM, whereas kempopeptin C (**3**) exhibited three-fold greater potency with IC_50_ values of ~0.3 µM (Table 2). The differences in potency could be attributed to the difference in the halogenation pattern between kempopeptins B and C (**2** and **3**).

In comparison with other structurally-related Ahp containing cyclic depsipeptides, featuring a basic residue on the *N*-terminal side of the Ahp moiety (Figure 1), kempopeptins B and C (**2** and **3**) were found to be less potent than cyanopeptolin 1020 (**4**) [27] against trypsin. Although a side by side comparison between kempopeptins and cyanopeptolin 1020 was not possible, it would be required to accurately quantify the differences in potency. Cyanopeptolin 1020 (**4**) is one of the most potent Ahp-containing trypsin inhibitors identified to date with an IC_50_ value of 670 pM [27]. The main structural differences between cyanopeptolin 1020 (**4**) and kempopeptins B and C (**2** and **3**) are the presence of Phe in place of Ile, Arg instead of Lys as the basic residue and a different side chain comprised of Glu and hexanoic acid. Furthermore, **4** lacks the OMe and halogen substitution in the Tyr residue. The differences in activity towards trypsin inhibition could be attributed to the presence of the Phe and possibly the side chain. This was based on previous docking studies of **4** and the structurally related inhibitor A90720A (**5**) onto bovine trypsin. Compound **5** shares the same scaffold as **4** apart from the replacement of Phe with the Leu moiety and a different side chain (Figure 1); however, it exhibited lower potency against trypsin inhibition (IC_50_ ~10 nM) [28]. It was proposed based on docking data that the presence of the bulky Phe residue results in favorable interaction and binding as it pushes the nearby *N*-Me-Tyr inward [27]. Despite the differences in potency towards trypsin between kempopeptins B and C (**2** and **3**) and cyanopeptolin 1020 (**4**), they exhibited comparable potency towards plasmin inhibition (**4**, IC_50_ ~ 0.49 µM) [27].

Symplocamide A (**6**) is another structurally-related Ahp-containing compound isolated from the marine cyanobacterium *Symploca* sp. [29] Compound **6** shares a similar scaffold as kempopeptins B and C (**2** and **3**) with minor differences. These include the presence of citrulline, a bulky but slightly basic moiety adjacent to the *N*-terminal end of Ahp, and Gln in place of Val in the side chain (Figure 1). Kempopeptins B and C (**2** and **3**) exhibited more potent trypsin inhibitory activity compared to symplocamide A (**6**, IC_50_ ~ 80 µM) [29]. Additionally, **2** and **3** exhibited more potent inhibitory activity than some of the structurally-related micropeptins (IC_50_ ~ 4–8 µM) against trypsin [30,31].

With respect to the activity and selectivity of kempopeptins B and C (**2** and **3**) against other serine proteases, the antiproteolytic activity of kempopeptin B (**2**) was previously evaluated against elastase and chymotrypsin [24]. Kempopeptin B (**2**) exhibited an IC_50_ value >67,000 nM against elastase and chymotrypsin and was found to be selective towards trypsin [24].

The antiproteolytic activity of many of the structurally-related analogues was not evaluated against serine proteases other than trypsin and chymotrypsin. Hence, there is lack of information about the overall specificity of this class of compounds and structure-activity relationship (SAR) data other than for trypsin. Additionally, the differences in enzyme inhibition assays carried out in different laboratories hinder accurate comparison and SAR conclusions.

### 2.3. Molecular Docking

**Figure 3:**
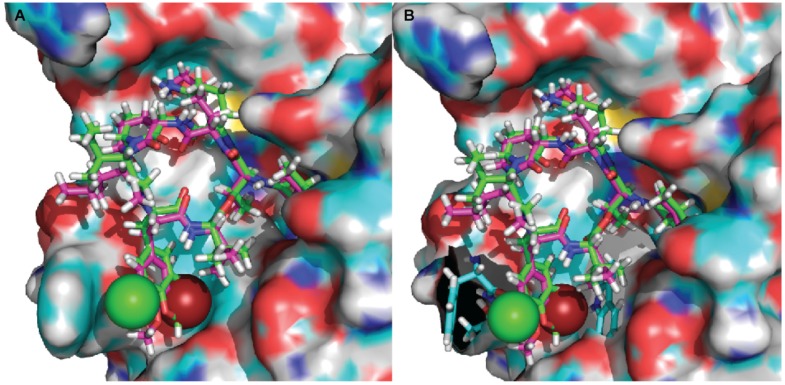
Docked structures of kempopeptin B (pink) and kempopeptin C (green) in matriptase. (**A**) The atomic sizes of the Br (red) and Cl (green) are displayed as spheres; (**B**) the residues of the matriptase enzyme in close proximity to Br (Trp) and Cl (Phe and Thr) are displayed in stick representation (cyan).

To better understand the binding mode of kempopeptins within the matriptase active site, molecular docking experiments were carried out using Glide-Dock (Maestro). The published crystal structure of matriptase (PDB: 2GV7) was used for docking. Kempopeptins B and C (**2** and **3**) were both docked into matriptase (PDB: 2GV7), and the interactions between the compound and the receptor binding pocket were examined (Figure 3). Our data show that both kempopeptins B and C (**2** and **3**) have the same binding pattern indicated by the overlaid structures of **2** and **3** (Figure 3). Compounds **2** and **3** were found to bind within the active site containing the catalytic triad (Ser 195, His 57 and Asp 102). Molecular modeling did not rationalize the differences in activity between kempopeptins B and C (**2** and **3**). Other factors such as the electronegativity and atomic size could affect the potency due to the differences in the halogen substituent between **2** and **3**. The differences in potency could be attributed to the atomic size where the larger Br atom, pointing towards the inner side of the pocket, might result in a steric hindrance compared to the Cl atom pointing outward (Figure 3A). The steric hindrance, due to the larger atomic size of Br, might result in pushing the molecule slightly away from the binding pocket resulting in lower binding affinity. Additionally, the electronic effects might also contribute to the differences in potency.

### 2.4. In Vitro Cleavage of CDCP1 by Matriptase

**Figure 4:**
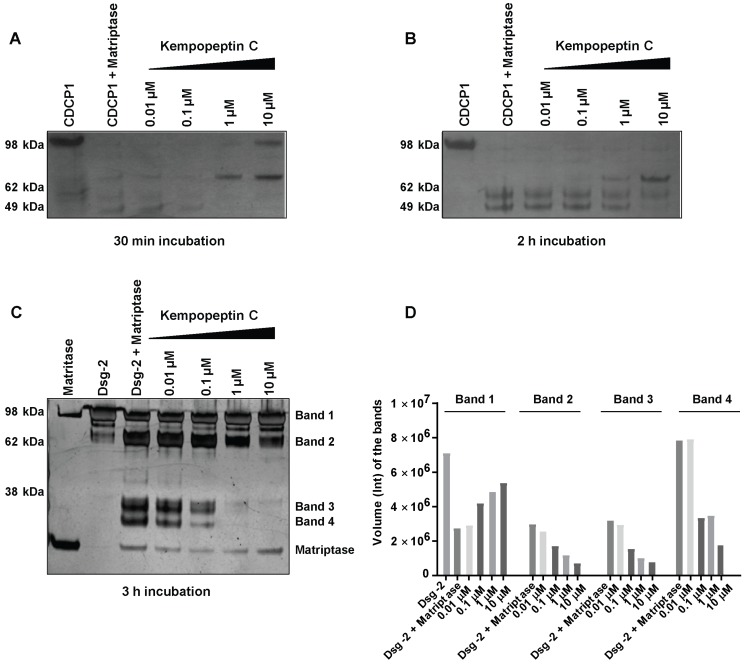
The effect of kempopeptin C (**3**) on the in vitro cleavage of CDCP1 and desmoglein-2 (Dsg-2) by matriptase. (**A**,**B**) Incubation of recombinant human CDCP1 with recombinant human matriptase (enzyme to substrate ratio 1:5) in 50 mM Tris, 50 mM NaCl, 0.01% Tween 20 (pH 9.0) at 37 °C for 30 min and 2 h in the presence and absence of different concentrations (10‒0.01 µM) of kempopeptin C (**3**). (**C**) Incubation of recombinant human Dsg-2 with recombinant human matriptase (enzyme to substrate ratio 1:5) in 50 mM Tris, 50 mM NaCl, 0.01% tween 20 (pH 9.0) at 37 °C for 3 h in the presence and absence of different concentrations (10‒0.01 µM) of kempopeptin C (**3**). Fragments were separated on SDS-PAGE under reducing conditions and silver stained. (**D**) Densitometric analysis of Dsg-2 bands in the SDS-PAGE in Figure 3C.

Due to the significance of these proteases in cancer progression and metastasis, as well as their functional redundancy with respect to targeting overlapping substrates, we focused our studies on examining the effect of kempopeptin C (**3**) on the downstream cellular substrates of matriptase. Due to the limited amount of **3**, our study aimed to provide a qualitative preliminary characterization of CDCP1 and Dsg-2 cleavage inhibition by kempopeptin C (**3**). CUB-domain-containing protein 1 (CDCP1), an integral membrane glycoprotein, has been shown to be upregulated in several cancers including breast, lung and colon cancer [32,33,34]. CDCP1 is considered a potential prognostic marker with expression levels found to correlate with the recurrence and survival rate of cancer patients [35,36]. Hence, CDCP1 is a potential therapeutic target supported by a study demonstrating the reduction in prostate cancer metastasis via the antibody-mediated inhibition of CDCP1 in vivo [37]. Apart from the full-length form (~135 kDa), a low molecular weight (LMW) *C*-terminal 70-kDa fragment was found to be endogenously expressed in several cancers [15]. The exact mechanism regulating the generation of the LMW fragment is poorly understood; however, there is evidence for the involvement of serine protease proteolytic activity [15,38]. Several studies provided evidence for serine protease-mediated (trypsin, plasmin and matriptase) cleavage of CDCP1 [15,38]. Therefore, we examined the ability of recombinant human matriptase to cleave CDCP1 in vitro and assessed the effect of kempopeptin C (**3**) on inhibiting/reducing this cleavage. Recombinant human matriptase was incubated with CDCP1 (final molar ratio 1:5) at different time points (30 min and 2 h) at 37 °C in the presence of varying concentrations of kempopeptin C (**3**) or solvent control (Figure 4A,B). The resulting protein fragments were separated by SDS-PAGE and subjected to silver staining. This incubation resulted in the cleavage of CDCP1 into LMW fragments with the full-length form not being detected. In the presence of kempopeptin C (**3**), matriptase cleaved CDCP1 at all concentrations tested, and **3** was able to only minimize the cleavage at 10 µM (indicated by the detection of the full length CDCP1) in a 30-min incubation period (Figure 4A). Furthermore, the 70-kDa LMW fragment was only detected in the presence of 10 and 1 µM of **3**, suggesting the extensive cleavage in the absence or presence of low concentrations of kempopeptin C (**3**). LMW fragments were more readily detected at longer incubation times (2 h).

### 2.5. In Vitro Cleavage of Desmoglein 2 by Matriptase

Among the earliest events in cancer metastasis is the loss of intracellular adhesion. Desmosomes are adhesive molecules localized at the cell membrane acting as an anchor for intermediate filaments in addition to being adhesive intracellular junctions [16]. Desmogleins (Dsg 1‒4) and desmocollins (Dsc 1‒3), comprising the core of the desmosomal adhesive complex, are glycoproteins and members of the cadherin superfamily of proteins [16,17]. Desmogleins have been reported to be aberrantly expressed in several cancers [39,40,41,42,43] and substrates of several proteases [14,40]. Dsg-2 is known to be a substrate of kallikrein 7 (KLK7) [40] and harbors a cleavage site for matriptase [14]. We therefore investigated the cleavage of Dsg-2 by matriptase in vitro and the ability of kempopeptin C (**3**) to inhibit or reduce this cleavage. Matriptase and Dsg-2 were incubated for 3 h at 37 °C in assay buffer (final molar ratio 1:5) in the presence of different concentrations of kempopeptin C (**3**) or solvent control (Figure 4C). The resulting protein fragments were separated by SDS-PAGE and subjected to silver staining. Incubating Dsg-2 with matriptase resulted in its cleavage into several low molecular weight fragments (~38 kDa). A slight dose-dependent reduction in the cleavage of Dsg-2 was noted upon the addition of the matriptase inhibitor, kempopeptin C (**3**), at 10 and 1 µM. The band intensities were quantified by densitometry, which revealed higher intensity of the full-length Dsg-2 (Band 1) in the presence of 10, 1 and 0.1 µM kempopeptin C (**3**) compared to lower concentrations of the inhibitor (**3**) (Figure 4D). Additionally, lower intensities of the lower molecular weight fragments (Bands 2–4) were noted in the presence of **3** at concentrations up to 0.1 µM. The incomplete cleavage of Dsg-2 by matriptase might be due to the relative affinity of kempopeptin C (**3**) to matriptase, such that it can block the cleavage of some low affinity bands, but have no effects on others. Additionally, the results of these in vitro experiments could vary by using different enzyme to substrate ratios, different time points and different pH, but the limited amount of kempopeptin C (**3**) hindered further optimization. This data suggest that Dsg-2 is indeed a substrate of matriptase [40], being cleaved and degraded, leading to potentially reduced cohesiveness and dissemination of tumor cells.

### 2.6. Level of Dsg-2 and CDCP1 in Breast Cancer Cell Lines

**Figure 5:**
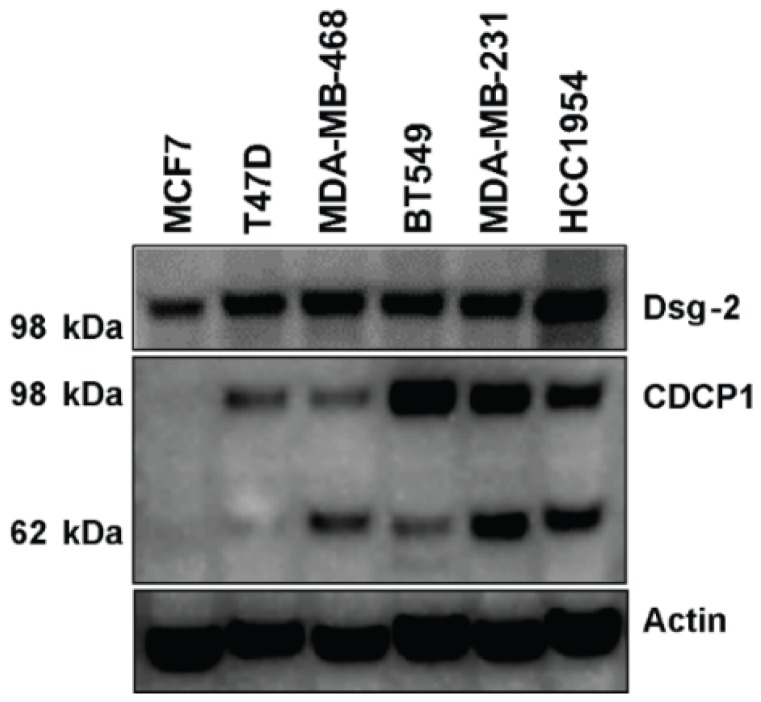
The expression level of Dsg-2 and CDCP1 in protein lysates collected from different breast cancer cell lines. The expression levels were assessed by Western blot probed with anti-Dsg-2 and anti-CDCP1 antibodies. Actin was used as the loading control.

Since Dsg-2 and CDCP1 are considered biomarkers of invasive forms of cancer and in order to choose a cell line to further investigate the effect of kempopeptins on the matriptase-dependent cleavage of Dsg-2 and CDCP1 in a cell-based system, we assessed the expression pattern of these substrates in a panel of breast cancer cell lines that differ in their hormonal status (Figure 5). Dsg-2 was found to be expressed in all cell lines tested; however, there were differences in the expression patterns of the full-length, as well as the low molecular weight CDCP1 fragment among the breast cancer cells tested. No CDCP1 was detected in the ER + MCF7, and the highest expression was noted in the triple negative (claudin low) breast cancer cells (BT549 and MDA-MB-231), as well as the HER2 + HCC1954. Additionally, T47D (ER+) was found to express only the full-length CDCP1.

### 2.7. Effects of Kempopeptins B and C on the Viability of MDA-MB-231

The effect of kempopeptins B and C (**2** and **3**) on the viability of cancer cells was examined using the MDA-MB-231 cell line. Both compounds (**2** and **3**) exhibited no effect on the growth at all concentrations tested with % viability greater than 90% (Supplementary Figure S1).

### 2.8. Effects of Kempopeptins B and C on the CDCP1 Cleavage in MDA-MB-231

**Figure 6:**
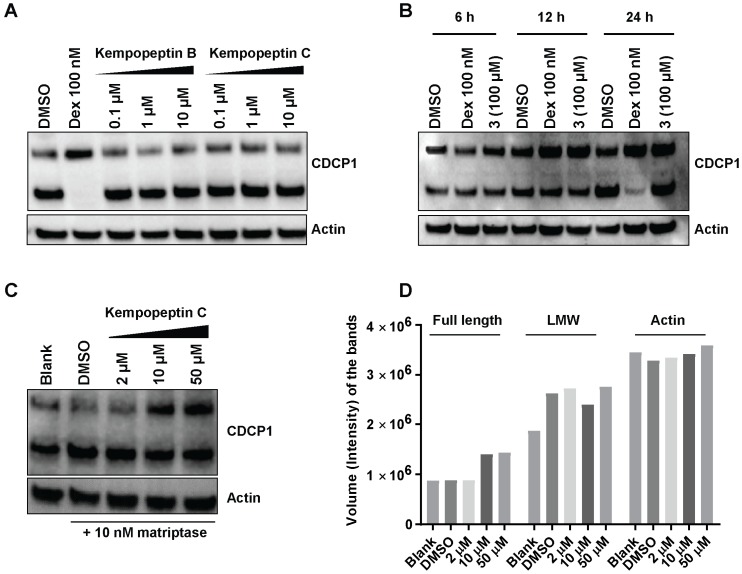
The effect of kempopeptins B and C (**2** and **3**) on CDCP1 cleavage in MDA-MB-231. (**A**) MDA-MB-231 cells were treated for 72 h with different concentrations of the test compounds. Following 72 h, the lysates were collected and analyzed by Western blot. (**B**) MDA-MB-231 cells were treated at different time points with 100 µM of kempopeptin C (**3**). Following 6 h, 12 h and 24 h, the lysates were collected and analyzed by Western blot. Dexamethasone (Dex) was used as a positive control. (**C**) MDA-MB-231 cells treated for 24 h with different concentrations of **3** in the presence of 10 nM recombinant human matriptase. Cell lysates were harvested and analyzed by Western blot. (**D**) Densitometric analysis of the full-length, LMW of CDCP1 and actin bands in (C).

Since kempopeptins B and C (**2** and **3**) were shown to inhibit matriptase, which is known to be a cellular processor of CDCP1, we investigated the ability of kempopeptins to inhibit the cleavage of CDCP1 in a cell-based system. The experiment was performed by treating MDA-MB-231 cells with different concentrations of either kempopeptin B (**2**) or C (**3**) for 72 h (Figure 6A). Dexamethasone (Dex) 100 nM was used as a positive control. A prior study has shown that Dex blocks the cleavage of the full-length CDCP1 through the upregulation of the serine protease inhibitor PAI-1 [18]. No inhibition of CDCP1 cleavage was observed under these conditions. Additionally, the experiment was performed using higher concentrations of kempopeptin C (100 µM) at different time points (6, 12 and 24 h), but no inhibition of CDCP1 cleavage was detected (Figure 6B). The data suggest the involvement of other proteases or factors regulating the cleavage of CDCP1 or a higher concentration of the inhibitor is required.

Since MDA-MB-231 cells were reported to exhibit low expression levels of matriptase [12], we examined the effect of kempopeptin C (**3**) on the cleavage of cellular CDCP1 using MDA-MB-231 supplemented with exogenous recombinant human matriptase (10 nM). In this experiment, we aimed to investigate the ability of **3** to inhibit cell-expressed CDCP1 rather than the purified recombinant protein in vitro. In contrast to the well-controlled in vitro assay, several factors might influence the extracellular matriptase activity in the cell-based assay; for example, the occurrence of additional secreted cellular factors, differences in pH, in addition to the unknown enzyme:substrate:inhibitor ratio. Following 24 h of incubation with different concentrations of **3** and 10 nM matriptase, our data show a slight dose-dependent reduction in the cleavage of the full-length form of the protein (Figure 6C). This was indicated by densitometric analysis (Figure 6D), which revealed an increase in the intensity of the band corresponding to the full-length CDCP1 in the presence of 50 and 10 µM of the inhibitor (**3**) while the intensity of the low molecular weight (LMW) fragment remained unchanged. The inability of kempopeptin C (**3**) to efficiently inhibit matriptase-induced cellular cleavage of CDCP1 could be cell type dependent, as matriptase may not proteolytically process CDCP1 in this cell line. This was supported by a study showing that a reduction in matriptase expression in PC3 and DU145 cells failed to show any changes in the level of the 70-kDa fragment [15]. Additionally, matriptase is one of at least two other proteases known to process CDCP1, where plasmin is the main protease cleaving CDCP1 in MDA-MB-231 cells [18]. Although plasmin and matriptase are equally inhibited by kempopeptin C (**3**) in vitro, the inhibition of plasmin might be different in the cellular studies. This could partly be due to the differences in plasmin levels affecting the enzyme to substrate ratio and the presence of other proteases, which might cleave plasmin and not be inhibited by **3**.

### 2.9. Effects of KempopeptinC on the Migration of MDA-MB-231

**Figure 7:**
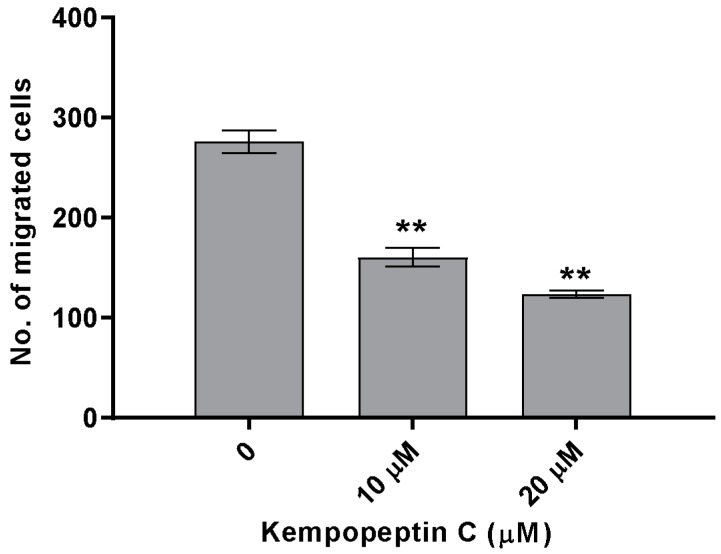
The effect of kempopeptin C (**3**) on the migration of MDA-MB-231. MDA-MB-231 were incubated for 48 h in the presence of different concentrations (0, 10 or 20 µM) of **3** and the effect was compared to the solvent control. The graph represents the number of migrated cells in each treatment group. The asterisks denote the significance of *p* ≤ 0.01 relative to the solvent control using the two-tailed unpaired *t*-test. Data are presented as the mean ± SD, *n* = 3.

The significance of our compounds in inhibiting matriptase and targeting its downstream cellular substrates was evaluated by examining the effect of kempopeptin C (**3**) on the migration of highly invasive breast cancer cells. The effect on migration was investigated by treating MDA-MB-231 cells with different concentrations of the test compound or solvent control for 48 h. Following 48 h, migrated cells were stained with crystal violet, and the number was counted under the microscope. Kempopeptin C (**3**) inhibited the migration of the invasive triple negative breast cancer cells with ~37% and 60% inhibition at 10 µM and 20 µM, respectively (Figure 7). To rule out that proteasome inhibition might be responsible for the migration inhibitory activity, the effect of kempopeptin C (**3**) on the trypsin-like activity of proteasomes was tested in vitro. Our data showed that kempopeptin C (**3**) did not inhibit the trypsin-like activity of proteasomes compared to the positive control epoxomicin (Supplementary Figure S2).

## 3. Experimental Section

### 3.1. General Experimental Procedures

^1^H and 2D NMR spectra for **3** in DMSO-*d*_6_ were recorded on a Bruker 600-MHz Avance II Spectrometer (Bruker Biospin Corporation, Billerica, MA, USA). The spectra were referenced using the residual solvent signal (*δ*_H/C_ 2.5/39.5 (DMSO-*d*_6_)). The HRESIMS data were obtained in the positive mode using the Agilent LC-TOF mass spectrometer (Agilent, Santa Clara, CA, USA) equipped with the APCI/ESI multimode ion source detector. The optical rotation was measured using a Perkin-Elmer 341 polarimeter (PerkinElmer Inc., Wellesley, MA, USA)

### 3.2. Biological Material

This sample was collected in 2009, and it was a recollection of the previous sample from Kemp Channel in Florida Keys identified as *Lyngbya* sp. [24].

### 3.3. Extraction and Isolation

The lyophilized material was extracted with 1:1 CH_2_Cl_2_:MeOH to yield 8.2 g of the nonpolar extract. The resulting lipophilic extract was partitioned between hexanes: 20% aqueous MeOH. The methanolic extract was singly concentrated and further partitioned between BuOH:H_2_O. The BuOH fraction (1.08 g) was further purified by silica column chromatography with increasing gradients of *i*-PrOH in CH_2_Cl_2_ (2%, 5%, 10%, 20%, 50% up to 100% *i*-PrOH) followed by 100% MeOH. The fraction from 50% *i*-PrOH was subjected to C18 SPE cleanup using increasing gradients of MeOH in H_2_O (25%, 50%, 75% and 100% MeOH). The 75% MeOH was further purified by semipreparative reversed-phase HPLC (YMC AQ, 250 × 10 mm, 5 μm) using a linear gradient of MeOH in H_2_O (50‒100% for 60 min, 100% for another 10 min) to afford kempopeptin A (1.1 mg, *t*_R_ 26.5 min).

The 100% MeOH fraction from silica column chromatography was subjected to further purification using the silica SPE column, eluting with increasing gradients of MeOH in CH_2_Cl_2_. The fraction eluting from 25% MeOH was further purified by semipreparative reversed-phase HPLC (Synergi Hydro-RP, 250 × 10 mm, 5 μm) using a linear gradient of MeOH-H_2_O (60–100% MeOH for 40 min, followed by 100% MeOH for 15 min) to afford kempopeptin C (0.5 mg, *t*_R_ 23.8 min).

Kempopeptin A: colorless, amorphous powder; ^1^H NMR is identical to that of the authentic sample (Supporting Information); Low resolution electrospray ionization mass spectrometry (LRESIMS) *m/z* 1013.5 [M + Na]^+^.

Kempopeptin C: colorless, amorphous powder; [α]${}_{20}^{D}$ − 37.3 (*c* 0.12, MeOH); ^1^H and ^13^C NMR data, Table 1; HRESIMS *m/z* 971.5006 [M + Na]^+^ (calcd. for C_46_H_73_^35^ClN_8_O_11_Na, 971.4985), *m/z* 973.5002 [M + Na]^+^ (calcd. for C_46_H_73_^37^ClN_8_O_11_Na, 973.4956).

Kempopeptin B, which was tested in parallel with kempopeptin C, was from a previous isolation of *Lyngbya* sp. collected from Kemp Channel in Florida Keys [24].

Kempopeptin B: colorless, amorphous powder; [α]${}_{20}^{D}$ − 17.1 (*c* 0.07, MeOH); ^1^H NMR (Supporting Information); LRESIMS *m/z* 993.4 [M + H]^+^ and *m/z* 995.4 [M + H]^+^ (1:1 ion cluster)

### 3.4. In Vitro Protease Inhibition Assays

The trypsin inhibition assay was carried out at room temperature by incubating 10 µL of trypsin from porcine pancreas (T0303, Sigma, St. Louis, MO, USA) (100 µM prepared in assay buffer) with 39 µL of assay buffer (50 mM Tris-HCl, 100 mM NaCl, 1 mM CaCl_2_; pH 7.8) in the presence of 1 µL of different concentrations of inhibitors (**2** and **3**; half log dilutions prepared in DMSO) or the solvent control. After a 30-min incubation time, 50 µL of the 1.5 mM substrate: *N*α-benzoyl-dl-arginine-4-nitroanilide (prepared as 2 mM stock solution in DMF and further diluted to 1.5 mM in assay buffer) were added to each well. The reaction was monitored by measuring the absorbance at 405 nm every 30 s for 30 min using SpectraMax M5 plate reader (Molecular Devices, Sunnyvale, CA, USA).

The plasmin inhibition assay was carried out by incubating 5 µL of 6.6 mM plasmin from human plasma (P1867, Sigma, St. Louis, MO, USA) with 1 µL of different concentrations of inhibitors (**2** and **3**; half log dilutions prepared in DMSO) or the solvent control in the presence of 89 µL assay buffer (0.05 M Tris-HCl, pH 8.9). After a 30-min incubation time, 5 µL of 1.7 mM substrate solution (*N*-p-Tosyl-d-Gly-Pro-Lys-4-nitroanilide acetate salt; prepared in assay buffer) were added to each well. The reaction was monitored by measuring the absorbance at 405 nm every 30 s for 30 min using the SpectraMax M5 plate reader (Molecular Devices).

In matriptase inhibition assay, 50 µL of recombinant human matriptase (R&D Systems, Minneapolis, MN, USA; 3946-SE-010) (3.8 mM prepared in assay buffer: 50 mM Tris, 50 mM NaCl, 0.01% Tween 20; pH 9.0) and 1 µL of different concentrations of inhibitors (**2** and **3**; half log dilutions prepared in DMSO) or the solvent control were pre-incubated at room temperature for 15 min. Following the incubation period, 50 µL of the 50 µM substrate solution (Boc-Gln-Ala-Arg-AMC) (R&D Systems, Minneapolis, MN, USA; ES014) were added to each well, and the enzyme activity was monitored by measuring the increase in fluorescence signal from the fluorescently-labeled substrate every 30 s for 30 min (Ex 380 nm, Em 460 nm) using the SpectraMax M5 plate reader (Molecular Devices).

### 3.5. Molecular Docking

The molecular docking experiments were carried out using Glide-Dock (Maestro from Schrödinger, Inc., New York, USA). Prior to docking, the ligand and receptor (PDB: 2GV7) were prepared using LigPrep and the Protein Preparation Wizard workflow, respectively. The grid generation was then followed, and the grid was prepared with a 20 Å cubic box in the receptor. The interactions of the docked structures of **2** and **3** with the receptor were examined and analyzed using Pymol software (Schrödinger, Inc., New York, USA).

### 3.6. In Vitro Cleavage of Desmoglein 2 and CDCP1 by Matriptase

Recombinant human matriptase (R&D Systems; 3946-SE-010) and recombinant human desmoglein 2 FC Chimera (R&D Systems; 947-DM-100) or recombinant protein of human CUB domain containing protein 1 (CDCP1), transcript variant 1 (OriGene; TP320633, Rockville, MD, USA) were incubated in an enzyme to substrate ratio (1:5) for 30 min, 1 h, 2 h or 3 h, pH 9.0, at 37 °C. Briefly, 50 µL of 154 µM matriptase were incubated with 50 µL of 225 µM Dsg-2 or 215 µM CDCP-1 to give final concentrations of 77 µM, 112.5 µM and 107.5 µM, respectively. The enzyme and substrate were both prepared in assay buffer (50 mM Tris, 50 mM NaCl, 0.01% Tween 20; pH 9.0). The solution was then treated with 1 µL of different concentrations of kempopeptin C (**3**) (prepared in DMSO) or solvent control. The total volume of the reaction was 100 µL. The reaction was then incubated for the indicated period of time at 37 °C. After the incubation period, the reaction was stopped by freezing. Twenty five microliters of each sample were then boiled for 5 min in 5 µL of sample buffer containing 2% mercaptoethanol. Dsg-2 and CDCP-1 fragments were then separated by SDS-PAGE (NuPAGE 4‒12% Bis-Tris mini gels, Invitrogen, Carlsbad, CA, USA) and stained using the silver stain kit (Pierce).

### 3.7. Cell Viability Assay

MDA-MB-231 cells were seeded in 96-well plates (10,000 cells/well). After 24 h of incubation, the cells were treated with different concentrations of kempopeptins B or C (**2** or **3**) or the solvent control (DMSO). Following 48 h of incubation, cell viability was measured using 3-(4,5-dimethylthiazol-2-yl)-2,5-diphenyltetrazolium bromide according to the manufacturer's instructions (Promega, Madison, WI, USA).

### 3.8. Expression Levels of Dsg-2 and CDCP1 in Breast Cancer Cell Lines

Cell lysates were collected from a panel of breast cancer cells with different hormonal status (MCF7, T47D, MDA-MB-468, BT549, MDA-MB-231 and HCC1954). Fifteen micrograms of proteins were separated using NuPAGE 4‒12% Bis-Tris mini gels, Invitrogen. The gel was transferred to polyvinylidene difluoride (PVDF) membranes, probed with Dsg-2 (R&D Systems; AF947), CDCP1 (Cell Signaling; 4115S) and actin (Cell Signaling; 4970S) antibodies, and detected with SuperSignal West Femto Maximum Sensitivity Substrate (Pierce). The secondary anti-rabbit (7074S) and anti-mouse (7076S) antibodies were obtained from Cell Signaling Technology (Danvers, MA, USA), and anti-goat antibody (HAF109) was obtained from R&D Systems.

### 3.9. Cleavage of CDCP1 in the MDA-MB-231 Cell Line

MDA-MB-231 cells (150,000 cells/well) were seeded in 12-well plates. After 24 h, the media were replaced, and cells were treated with different concentrations of inhibitors (**2** or **3**; prepared in DMSO), the solvent control or the positive control dexamethasone. Whole-cell protein lysates were collected after 6, 12, 24 and 72 h using PhosphoSafe lysis buffer (Novagen, Madison, USA) and the protein concentration measured using the BCA Protein Assay kit (Pierce; 23227; Thermofisher Scientific; Waltham, MA, USA). Twenty micrograms of protein were separated by SDS-PAGE, transferred to polyvinylidene difluoride (PVDF) membranes, probed with CDCP1 antibody (Cell Signaling; 4115S) and detected with SuperSignal West Femto Maximum Sensitivity Substrate (Pierce).

In experiments where matriptase was added to the cells, MDA-MB-231 cells were seeded in 12-well plates using serum containing DMEM. Twenty four hours later, the media was replaced with serum-free DMEM, and the cells were starved for 24 h. After 24 h, the cells were washed with PBS and fresh serum-free medium containing 10 nM matriptase (R&D Systems; 3946-SE-010) in the presence of different concentrations of kempopeptin C (**3**) (prepared in DMSO) or the solvent control was added. The cells were then incubated for 1 h before being lysed for Western blot analysis.

### 3.10. Transwell Migration Assays

The migration assays were performed using BD falcon cell culture inserts (353097; PET membrane, 8-µm pore size; Fisher Scientific; Waltham, MA, USA) in 24-well plates. In brief, 5000 cells of MDA-MB-231 were suspended in DMEM containing 10% FBS, treated with either different concentrations of kempopeptins C (**3**; prepared in DMSO) or the solvent control. Five hundred microliters were seeded into each insert, and 750 µL of complete medium treated with the same concentration of the compounds were seeded on the other side of the membrane. The plate was then incubated for 48 h (5% CO_2_, 37 °C). At the end of the incubation period, non-migrated cells were removed using cotton swabs and kimwipes, and migrated cells (to the underside of the membrane) were stained with crystal violet and the number enumerated under the microscope. The assays were performed in triplicate.

### 3.11. Proteasome Activity Assay

The inhibition of proteasomes was assessed using the Proteasome Glo Trypsin-Like Activity Assay from Promega according to the manufacturer’s instructions. Briefly, in white 96-well plates, kempopeptin C (**3**) (10 and 1 µM final concentration) was incubated at room temperature with 0.1 µg 20S proteasome (Enzo Life Sciences, Farmingdale, NY, USA) for 10 min to allow enzyme inhibitor interactions. Following the incubation period, the detection reagent containing Z-LRR-aminoluciferin substrate was added. The plate was gently mixed at 400 rpm for 30 s and then incubated at room temperature for 10 min. The luminescence signal was then measured using the EnVision plate reader (PerkinElmer, Waltham, MA, USA). Epoxomicin was used as a positive control.

## 4. Conclusions

The implication of the dysregulated activity of serine proteases in cancer progression has been extensively studied. The discovery of kempopeptin C (**3**), a novel chlorinated analogue of kempopeptin B (**2**), adds to the family of Ahp-containing cyclic depsipeptides from marine cyanobacteria. This class of cyanobacterial secondary metabolites has been shown to inhibit serine proteases with different selectivity profiles. We have demonstrated the ability of kempopeptins B and C (**2** and **3**), featuring a basic residue at the *N*-terminal end of Ahp, to inhibit trypsin, plasmin and matriptase. The biological activity of most of the compounds belonging to this class of cyanobacterial secondary metabolites has not been evaluated beyond the classical enzyme inhibition assays. Due to the significance of these proteases in cancer progression and metastasis, we investigated the ability of the matriptase inhibitor kempopeptins C (**3**) to target the downstream cellular substrates Dsg-2 and CDCP1. Additionally, we have shown that blocking the cleavage of the adhesive molecules comprising the intracellular junctions, by **3**, was able to inhibit the migration of the highly-invasive breast cancer cells. This class of inhibitors could be developed as chemical probes to understand the role of different proteases in cancer progression.

## Figures and Tables

**Table 1:** NMR spectral data for kempopeptin C (**3**) at 600 MHz (^1^H), 150 MHz (^13^C) in DMSO-*d*_6._

Unit	C/H no.	*δ*_H_ (*J* in Hz)	*δ*_C_, mult *^a^*	COSY	HMBC
Val-1	1		172.4, qC		
	2	4.66, dd (9.2, 5)	56.0, CH	NH, H-3	1, 5, 1 (*N*,*O*-diMe-Cl-Tyr)
	3	2.06, m	30.3, CH	H-2, H_3_-4, H_3_-5	5
	4	0.87, d (6.9)	19.0, CH_3_	H-3	3
	5	0.76, d (6.9)	17.3, CH_3_	H-3	2, 3
	NH	7.69, d (9.4)		H-2	1 (*N*,*O*-diMe-Cl-Tyr)
*N*,*O*-diMe-Cl-Tyr	1		169.2, qC		
	2	5.05, dd (11.7, 2.2)	60.1, CH	H-3a, H-3b	
	3a	3.22, dd (−14, 2.2)	32.6, CH_2_	H-2, H-3b	4, 9
	3b	2.80, dd (−14, 11.7)		H-2, H-3a	4, 9
	4		130.7, qC		
	5	7.25 d (1.5)	130.4, CH		6, 7
	6		121.4, qC		
	7		153.6, C		
	8	7.06, d (8.5)	113.0, CH	H-9	3, 6, 7
	9	7.14, dd (8.6, 1.5)	129.2, CH	H-8	3, 7
	O-Me	3.78, s	56.0, CH_3_		7
	N-Me	2.74, s	29.9, CH_3_		2, 1 (Ile)
Ile	1		169.7, qC		
	2	4.38, d (11)	53.9, CH		1
	3	1.82, m	32.6, CH	H-6	
	4a	1.11, m	23.4, CH_2_	H-4b, H-5	5
	4b	0.65, m		H-4a	
	5	0.62, *t* (6.5)	9.86, CH_3_	H-4a	3, 4
	6	−0.14, d (6.5)	13.4, CH_3_	H-3	2, 3, 4
Ahp	2		169.8, qC		
	3	4.45, m	48.6, CH	NH, H-4a, H-4b	2, 6
	4a	2.58, m	21.4, CH_2_	H-3, H-4b	
	4b	1.74, m		H-3, H-4a	
	5	1.76, m	29.4, CH_2_	H-6	
	6	4.93, br s	73.6, CH	H-5, 6-OH	
	6-OH	6.17, d (2.7)		H-6	
	NH	7.35, d (9.4)		H-3	1 (Lys)
Lys	1		170.3, qC		
	2	4.28, br	51.5, CH	NH, H-3b	
	3a	2.03, m	29.0, CH_2_	H-3b, H-5	
	3b	1.42, m		H-2, H-3a	
	4	1.26, m	21.9, CH_2_	H-3a	
	5	1.49, m	25.9, CH_2_	H-6	
	6	2.73, m	38.4, CH_2_	H-5, NH_2_	
	NH	8.45, d (8.5)		H-2	1 (Thr)
	NH_2_	7.63, br s		H-6	
Thr	1		169.2, qC		
	2	4.64, d (9.2)	54.5, CH	NH, H-3	1, 1 (Val-1)
	3	5.51, q (6.9)	71.5, CH	H-2, H_3_-4	4, 1 (Val-1)
	4	1.22, d (6.5)	17.3, CH_3_	H-3	3
	NH	7.83, d (7.7)		H-2	1 (Val-2)
Val-2	1		172.4, qC		
	2	4.34, d (8.0)	57.3, CH	NH, H-3	1, 4, 5
	3	2.03, m	29.8, CH	H-2, H-5	
	4	0.87, d (6.8)	19.0, CH_3_		
	5	0.85, d (6.8)	17.8, CH_3_	H-3	
	NH	7.84, d (8.1)		H-2	1, 1 (Ba)
Ba	1		172.3, qC		
	2	2.17, m	36.7, CH_2_	H-3	1, 4
	3	1.52, m	18.5, CH_2_	H-2, H_3_-4	1, 4
	4	0.86, *t* (7.5)	13.2, CH_3_	H-3	

*^a^* Multiplicity derived from the HSQC spectrum.

**Table 2:** IC_50_ values of kempopeptins B (**2**) and C (**3**) against serine proteases.

Compound	Trypsin	Plasmin	Matriptase
Kempopeptin B (**2**)	1.49 ± 0.18 µM	1.05 ± 0.19 µM	1.83 ± 0.19 µM
Kempopeptin C (**3**)	0.19 ± 0.05 µM	0.36 ± 0.09 µM	0.28 ± 0.03 µM

Data are presented as the mean ± SD, *n* = 3.

## Supplementary Materials

The following are available online at www.mdpi.com/1660-3397/15/9/290/s1, ^1^H NMR, COSY, TOCSY, HSQC and HMBC data for kempopeptin C (**3**); ^1^H NMR spectra for kempopeptins A (**1**) and B (**2**); comparison of ^1^H NMR spectra of kempopeptins B (**2**) and C (**3**) in DMSO-*d*_6_; cell viability assay data (Figure S1); proteasome trypsin-like activity assay (Figure S2).

## References

[1] Siegel R.L., Miller K.D., Jemal A. (2017). Cancer Statistics. CA Cancer J. Clin..

[2] Steeg P.S. (2016). Targeting metastasis. Nat. Rev. Cancer.

[3] Netzel-arnett S., Hooper J.D., Szabo R., Madison E.L., Quigley J.P., Bugge H., Antalis T.M. (2003). Membrane anchored serine proteases: A rapidly expanding group of cell surface proteolytic enzymes with potential roles in cancer. Cancer Metastasis Rev..

[4] Webb S.L., Sanders A.J., Mason M.D., Jiang W.G. (2011). Type II transmembrane serine protease (TTSP) deregulation in cancer. Front. Biosci..

[5] Shi Y.E., Torri J., Yieh L., Wellstein A., Lippman M.E., Dickson R.B. (1993). Identification and characterization of a novel matrix-degrading protease from hormone-dependent human breast cancer cells. Cancer Res..

[6] Bhatt A.S., Erdjument-Bromage H., Tempst P., Craik C.S., Moasser M.M. (2005). Adhesion signaling by a novel mitotic substrate of src kinases. Oncogene.

[7] Uhland K. (2006). Matriptase and its putative role in cancer. Cell. Mol. Life Sci..

[8] List K. (2009). Matriptase: A culprit in cancer?. Future Oncol..

[9] Ustach C.V., Huang W., Conley-LaComb M.K., Lin C.Y., Che M., Abrams J., Kim H.R.C. (2010). A novel signaling axis of matriptase/PDGF-D/β-PDGFR in human prostate cancer. Cancer Res..

[10] Lee S.L., Dickson R.B., Lin C.Y. (2000). Activation of hepatocyte growth factor and urokinase/plasminogen activator by matriptase, an epithelial membrane serine protease. J. Biol. Chem..

[11] Benaud C.M., Oberst M., Dickson R.B., Lin C.Y. (2002). Deregulated activation of matriptase in breast cancer cells. Clin. Exp. Metastasis.

[12] Welman A., Sproul D., Mullen P., Muir M., Kinnaird A.R., Harrison D.J., Faratian D., Brunton V.G., Frame M.C. (2012). Diversity of matriptase expression level and function in breast cancer. PLoS ONE.

[13] Jin J.S., Cheng T.F., Tsai W.C., Sheu L.F., Chiang H., Yu C.P. (2007). Expression of the serine protease, matriptase, in breast ductal carcinoma of Chinese women: Correlation with clinicopathological parameters. Histol. Histopathol..

[14] Wadhawan V., Kolhe Y.A., Sangith N., Gautam A.K.S., Venkatraman P. (2012). From prediction to experimental validation: Desmoglein 2 is a functionally relevant substrate of matriptase in epithelial cells and their reciprocal relationship is important for cell adhesion. Biochem. J..

[15] He Y., Wortmann A., Burke L.J., Reid J.C., Adams M.N., Abdul-Jabbar I., Quigley J.P., Leduc R., Kirchhofer D., Hooper J.D. (2010). Proteolysis-induced *N*-terminal ectodomain shedding of the integral membrane glycoprotein CUB domain-containing protein 1 (CDCP1) is accompanied by tyrosine phosphorylation of its *C*-terminal domain and recruitment of Src and PKC?. J. Biol. Chem..

[16] Green K.J., Gaudry C.A. (2000). Are desmosomes more than tethers for intermediate filaments?. Nat. Rev. Mol. Cell Biol..

[17] Kowalczyk A.P., Bornslaeger E.A., Norvell S.M., Palka H.L., Green K.J. (1999). Desmosomes: Intercellular adhesive junctions specialized for attachment of intermediate filaments. Int. Rev. Cytol..

[18] Law M.E., Corsino P.E., Jahn S.C., Davis B.J., Chen S., Patel B., Pham K., Lu J., Sheppard B., Nørgaard P. (2013). Glucocorticoids and histone deacetylase inhibitors cooperate to block the invasiveness of basal-like breast cancer cells through novel mechanisms. Oncogene.

[19] Galkin A.V., Mullen L., Fox W.D., Brown J., Duncan D., Moreno O., Madison E.L., Agus D.B. (2004). CVS-3983, a selective matriptase inhibitor, suppresses the growth of androgen independent prostate tumor xenografts. Prostate.

[20] Li P., Jiang S., Lee S.L., Lin C.Y., Johnson M.D., Dickson R.B., Michejda C.J., Roller P.P. (2007). Design and synthesis of novel and potent inhibitors of the type II transmembrane serine protease, matriptase, based upon the sunflower trypsin inhibitor-1. J. Med. Chem..

[21] Farady C.J., Sun J., Darragh M.R., Miller S.M., Craik C.S. (2007). The mechanism of inhibition of antibody-based inhibitors of membrane-type serine protease 1 (MT-SP1). J. Mol. Biol..

[22] Kwan J.C., Taori K., Paul V.J., Luesch H. (2009). Lyngbyastatins 8–10, elastase inhibitors with cyclic depsipeptide scaffolds isolated from the marine cyanobacterium *Lyngbya semiplena*. Mar. Drugs.

[23] Taori K., Matthew S., Rocca J.R., Paul V.J., Luesch H. (2007). Lyngbyastatins 5–7, potent elastase inhibitors from Floridian marine cyanobacteria, *Lyngbya* spp.. J. Nat. Prod..

[24] Taori K., Paul V.J., Luesch H. (2008). Kempopeptins A and B, serine protease inhibitors with different selectivity profiles from a marine cyanobacterium, *Lyngbya* sp.. J. Nat. Prod..

[25] Salvador L.A., Taori K., Biggs J.S., Jakoncic J., Ostrov D.A., Paul V.J., Luesch H. (2013). Potent elastase inhibitors from cyanobacteria: Structural basis and mechanisms mediating cytoprotective and anti-inflammatory effects in bronchial epithelial cells. J. Med. Chem..

[26] Lee A.Y., Smitka T.A., Bonjouklian R. (1994). Atomic structure of the trypsin-A90720A complex: A unified approach to structure and function. Chem. Biol..

[27] Gademann K., Portmann C., Blom J.F., Zeder M., Jüttner F. (2010). Multiple toxin production in the cyanobacterium *microcystis*: Isolation of the toxic protease inhibitor cyanopeptolin 1020. J. Nat. Prod..

[28] Bonjouklian R., Smitka T.A., Hunt A.H., Occolowitz J.L., Perun T.J., Doolin L., Stevenson S., Knauss L., Wijayaratne R., Szewczyk S. (1996). A90720A, a serine protease inhibitor isolated from a terrestrial blue-green alga *Microchaete loktakensis*. Tetrahedron.

[29] Linington R.G., Edwards D.J., Shuman C.F., McPhail K.L., Matainaho T., Gerwick W.H. (2008). Symplocamide A, a potent cytotoxin and chymotrypsin inhibitor from the marine Cyanobacterium *Symploca* sp.. J. Nat. Prod..

[30] Ploutno A., Shoshan M., Carmeli S. (2002). Three Novel Protease Inhibitors from a Natural Bloom of the Cyanobacterium *Microcystis aeruginosa*. J. Nat. Prod..

[31] Reshef V., Carmeli S. (2001). Protease inhibitors from a water bloom of the cyanobacterium *Microcystis aeruginosa*. Tetrahedron.

[32] Scherl-Mostageer M., Sommergruber W., Abseher R., Hauptmann R., Ambros P., Schweifer N. (2001). Identification of a novel gene, CDCP1, overexpressed in human colorectal cancer. Oncogene.

[33] Bühring H.J., Kuçi S., Conze T., Rathke G., Bartolović K., Grünebach F., Scherl-Mostageer M., Brümmendorf T.H., Schweifer N., Lammers R. (2004). CDCP1 identifies a broad spectrum of normal and malignant stem/progenitor cell subsets of hematopoietic and nonhematopoietic origin. Stem Cells.

[34] Ikeda J., Morii E., Kimura H., Tomita Y., Takakuwa T., Hasegawa J., Kim Y.K., Miyoshi Y., Noguchi S., Nishida T. (2006). Epigenetic regulation of the expression of the novel stem cell marker CDCP1 in cancer cells. J. Pathol..

[35] Awakura Y., Nakamura E., Takahashi T., Kotani H., Mikami Y., Kadowaki T., Myoumoto A., Akiyama H., Ito N., Kamoto T. (2008). Microarray-based identification of CUB-domain containing protein 1 as a potential prognostic marker in conventional renal cell carcinoma. J. Cancer Res. Clin. Oncol..

[36] Ikeda J., Oda T., Inoue M., Uekita T., Sakai R., Okumura M., Aozasa K., Morii E. (2009). Expression of CUB domain containing protein (CDCP1) is correlated with prognosis and survival of patients with adenocarcinoma of lung. Cancer Sci..

[37] Deryugina E.I., Conn E.M., Wortmann A., Partridge J.J., Kupriyanova T.A., Ardi V.C., Hooper J.D., Quigley J.P. (2009). Functional role of cell surface CUB domain-containing protein 1 in tumor cell dissemination. Mol. Cancer Res..

[38] Brown T.A., Yang T.M., Zaitsevskaia T., Xia Y., Dunn C.A., Sigle R.O., Knudsen B., Carter W.G. (2004). Adhesion or plasmin regulates tyrosine phosphorylation of a novel membrane glycoprotein p80/gp140/CUB domain-containing protein 1 in epithelia. J. Biol. Chem..

[39] Davies E., Cochrane R., Hiscox S., Jiang W., Sweetland H., Mansel R. (1997). The role of desmoglein 2 and E-cadherin in the invasion and motility of human breast cancer cells. Int. J. Oncol..

[40] Ramani V.C., Hennings L., Haun R.S. (2008). Desmoglein 2 is a substrate of kallikrein 7 in pancreatic cancer. BMC Cancer.

[41] Brennan D., Mahoney M.G. (2009). Increased expression of Dsg2 in malignant skin carcinomas: A tissue-microarray based study. Cell Adhes. Migr..

[42] Peitsch W.K., Doerflinger Y., Fischer-Colbrie R., Huck V., Bauer A.T., Utikal J., Goerdt S., Schneider S.W. (2014). Desmoglein 2 depletion leads to increased migration and upregulation of the chemoattractant secretoneurin in melanoma cells. PLoS ONE.

[43] Ormanns S., Altendorf-Hofmann A., Jackstadt R., Horst D., Assmann G., Zhao Y., Bruns C., Kirchner T., Knösel T. (2015). Desmogleins as prognostic biomarkers in resected pancreatic ductal adenocarcinoma. Br. J. Cancer.

