# Supplementary material for: Kempopeptin C, a Novel Marine-Derived Serine Protease Inhibitor Targeting Invasive Breast Cancer
Source: Mar Drugs. 2017 Sep 16;15(9):290. doi: 10.3390/md15090290 (PMC5618429; doi:10.3390/md15090290)
Supplement: Supplementary file 1 [file marinedrugs-15-00290-s001.pdf]

Supporting Information

Kempopeptin C, A Novel Marine-Derived Serine Protease Inhibitor Targeting Invasive Breast Cancer

Fatma H. Al-Awadhi [1,2], Lilibeth A. Salvador [3], Brian K. Law [2,4], Valerie J. Paul [5] and Hendrik Luesch [1,2,]*

[1]*Department of Medicinal Chemistry,* [2]*Center for Natural Products, Drug Discovery and Development (CNPD3), College of Pharmacy, University of Florida, Gainesville, FL.* [3]*Marine Science Institute, College of Science, University of the Philippines, Diliman, Quezon City, 1100, Philippines.* [4]*Department of Pharmacology and Therapeutics, College of Medicine, University of Florida, Gainesville, FL* [5]*Smithsonian Marine Station, 701 Seaway Drive, Fort Pierce, Florida, USA.*

**Contents**	**Page Number**
^{1}H NMR Spectrum of Kempopeptin C (**3**) in DMSO-d_6	S2
COSY Spectrum of Kempopeptin C (**3**) in DMSO-d_6	S3
TOCSY Spectrum of Kempopeptin C (**3**) in DMSO-d_6	S4
HSQC Spectrum of Kempopeptin C (**3**) in DMSO-d_6	S5
HMBC Spectrum of Kempopeptin C (**3**) in DMSO-d_6	S6
^{1}H NMR Spectrum of Kempopeptin A (**1**) in DMSO-d_6	S7
^{1}H NMR Spectrum of Kempopeptins B (**2**) in DMSO-d_6	S8
Comparison of ^{1}H NMR Spectra of Kempopeptins B (**2**) and C (**3**) in DMSO-d_6	S9
Figure S1. Effect of Kempopeptins B (**2**) and C (**3**) on Cell Viability	S10
Figure S2. Effect of Kempopeptin C (**3**) on Proteasome Activity	S11

^{1}H NMR spectrum of kempopeptin C (**3**) in DMSO-d_6 (600 MHz)

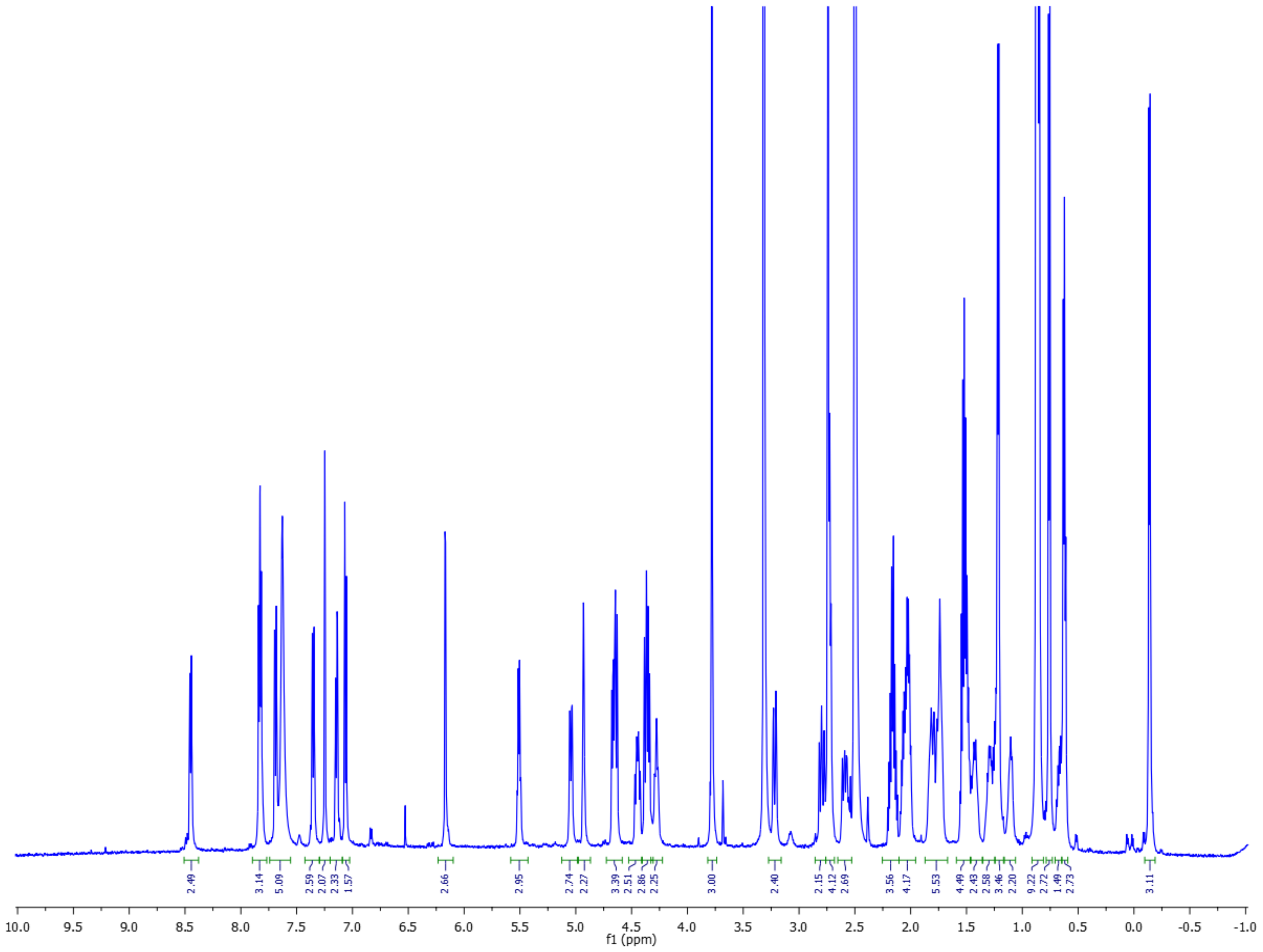

COSY spectrum of kempopeptin C (**3**) in DMSO-d_6 (600 MHz)

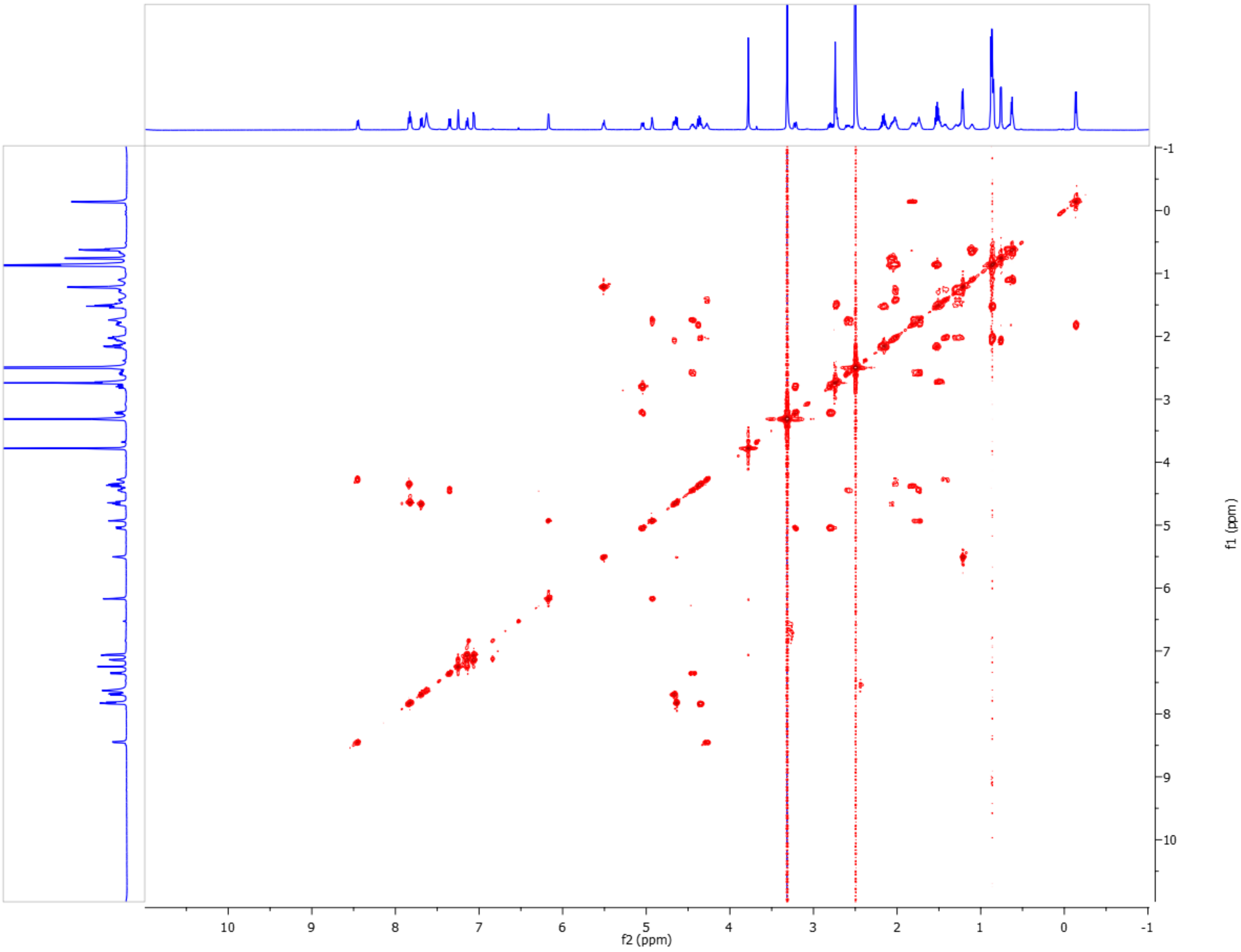

TOCSY spectrum of kempopeptin C (**3**) in DMSO-d_6 (600 MHz)

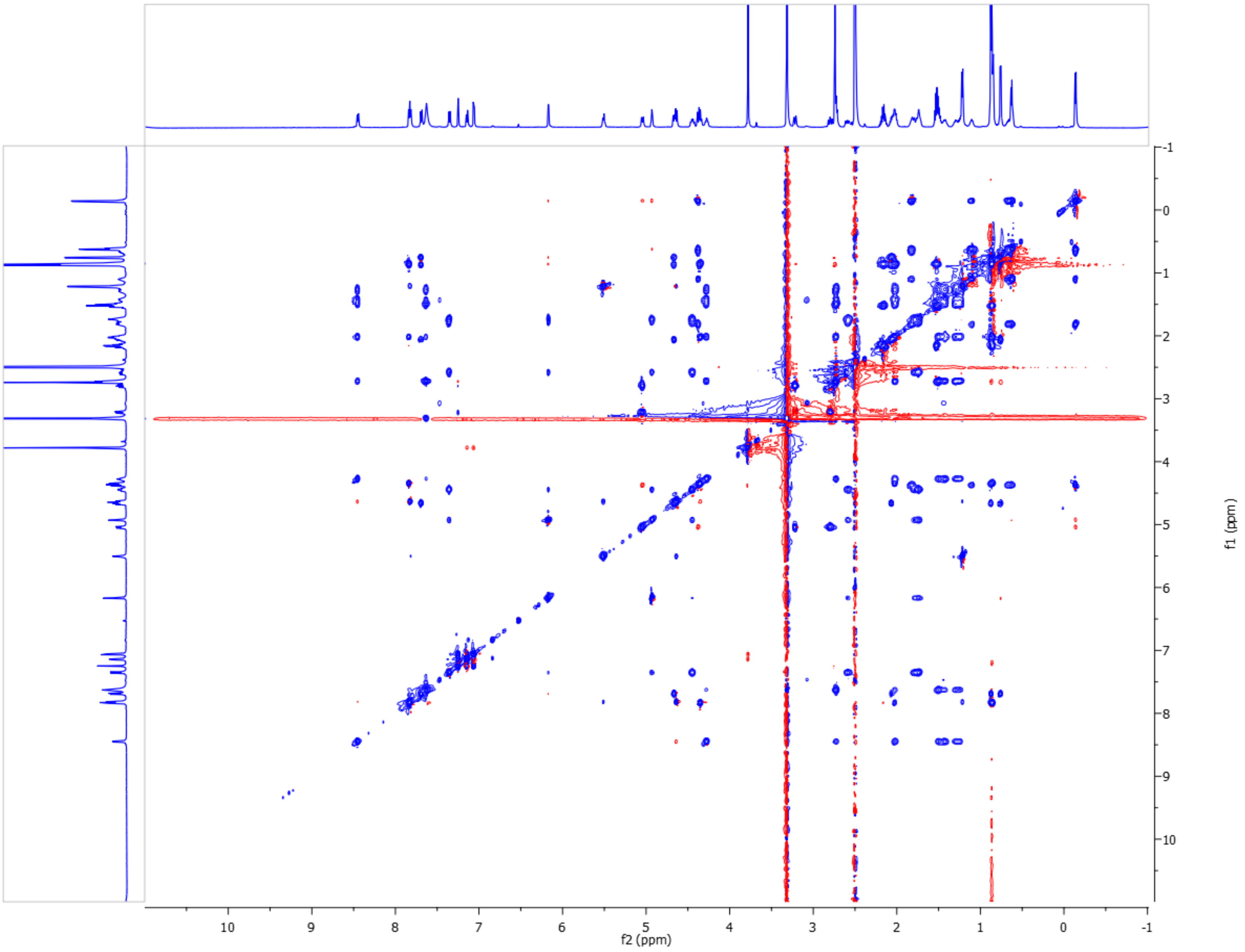

HSQC spectrum of kempopeptin C (**3**) in DMSO-d_6 (600 MHz)

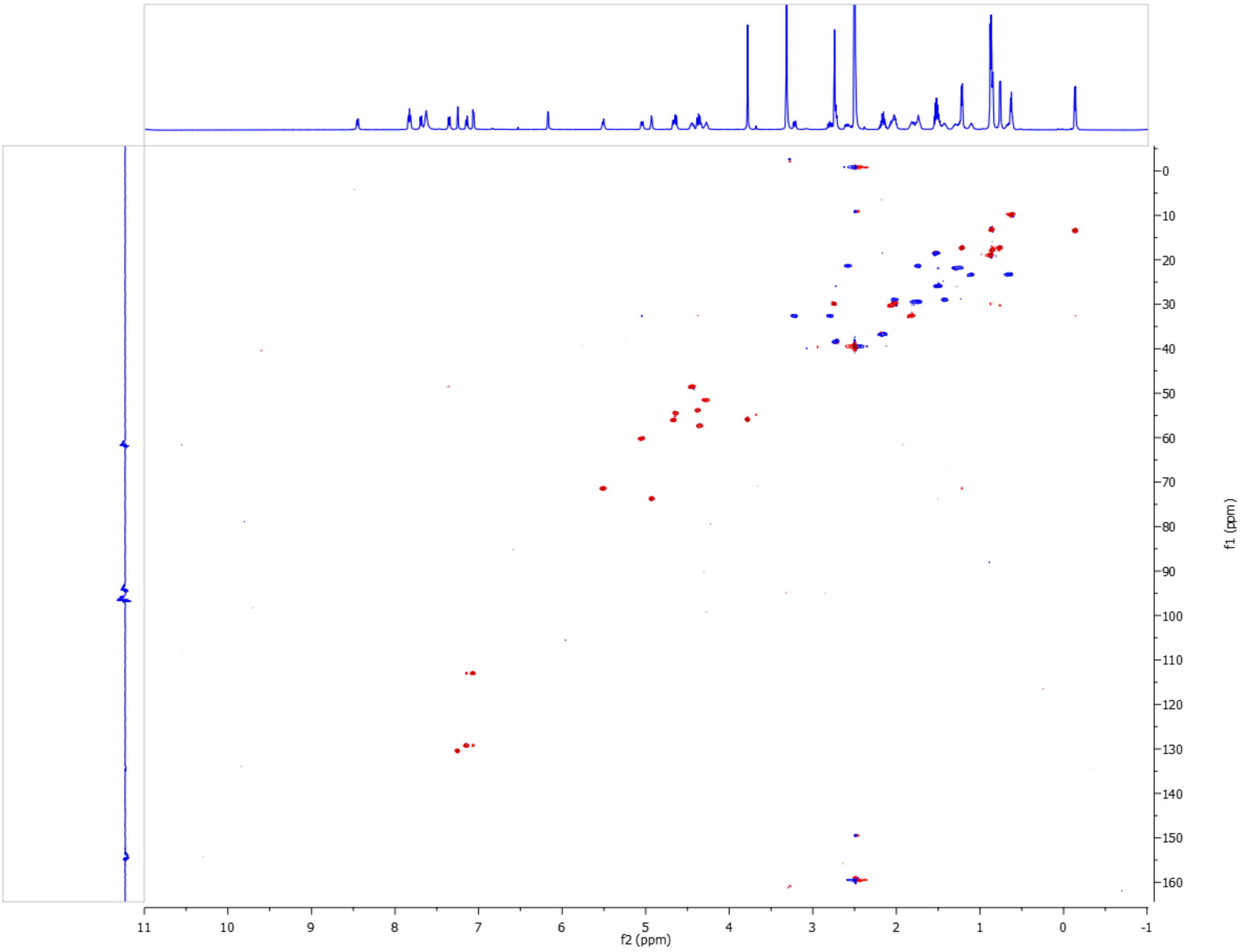

HMBC spectrum of kempopeptin C (**3**) in DMSO-d_6 (600 MHz)

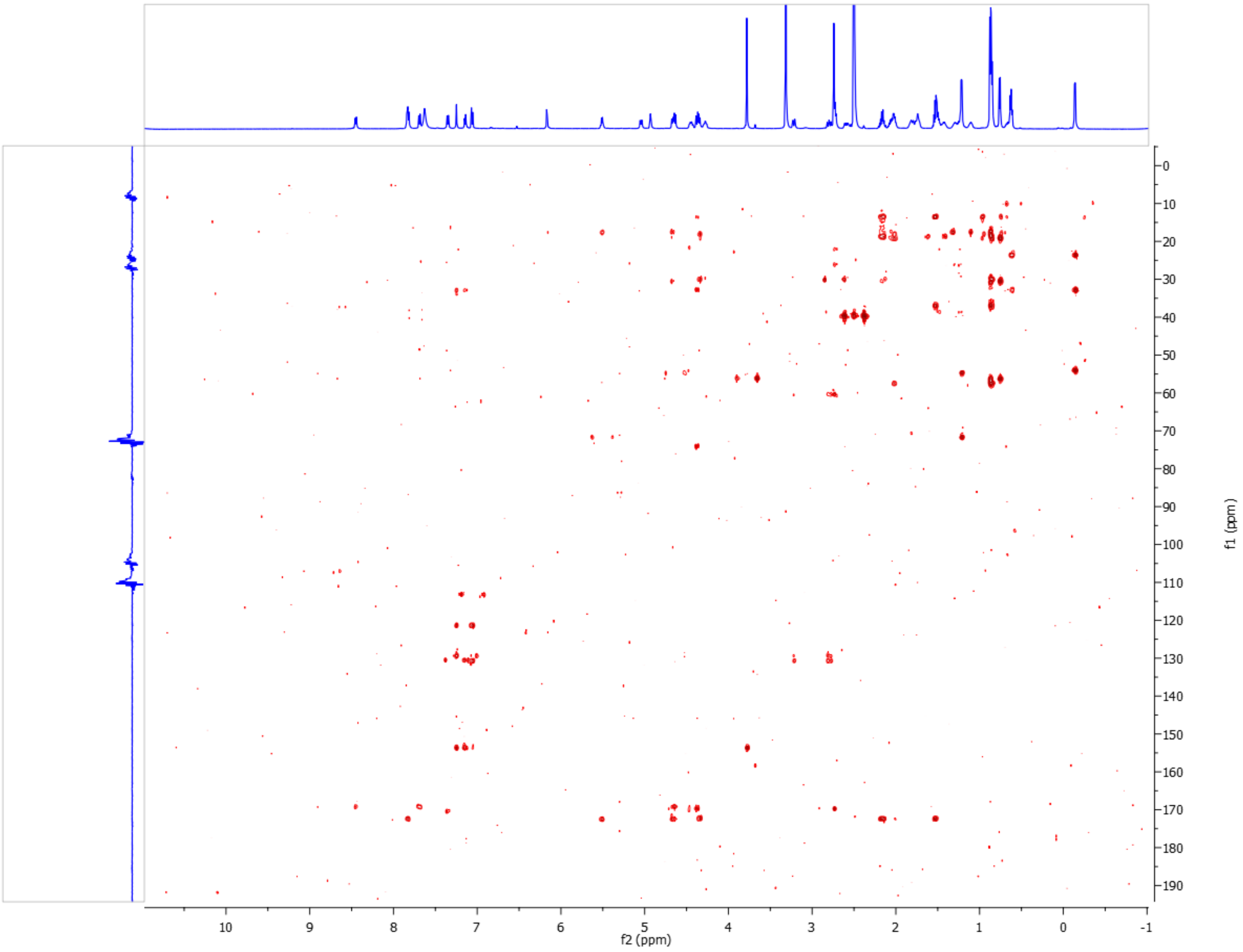

^{1}H NMR spectrum of kempopeptin A (**1**) in DMSO-d_6 (400 MHz)

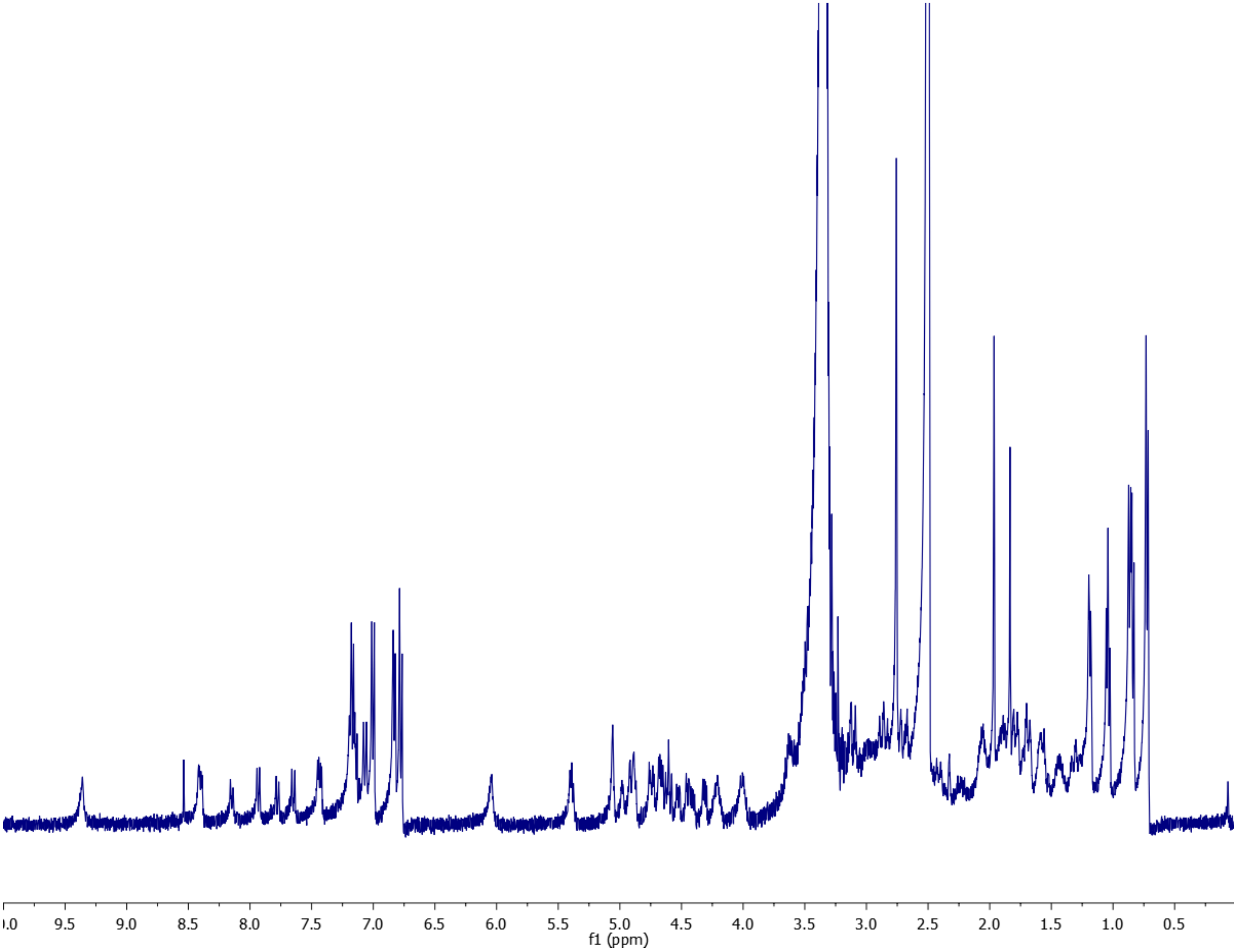

^{1}H NMR spectra of kempopeptins B (**2**) in DMSO-d_6 (600 MHz)

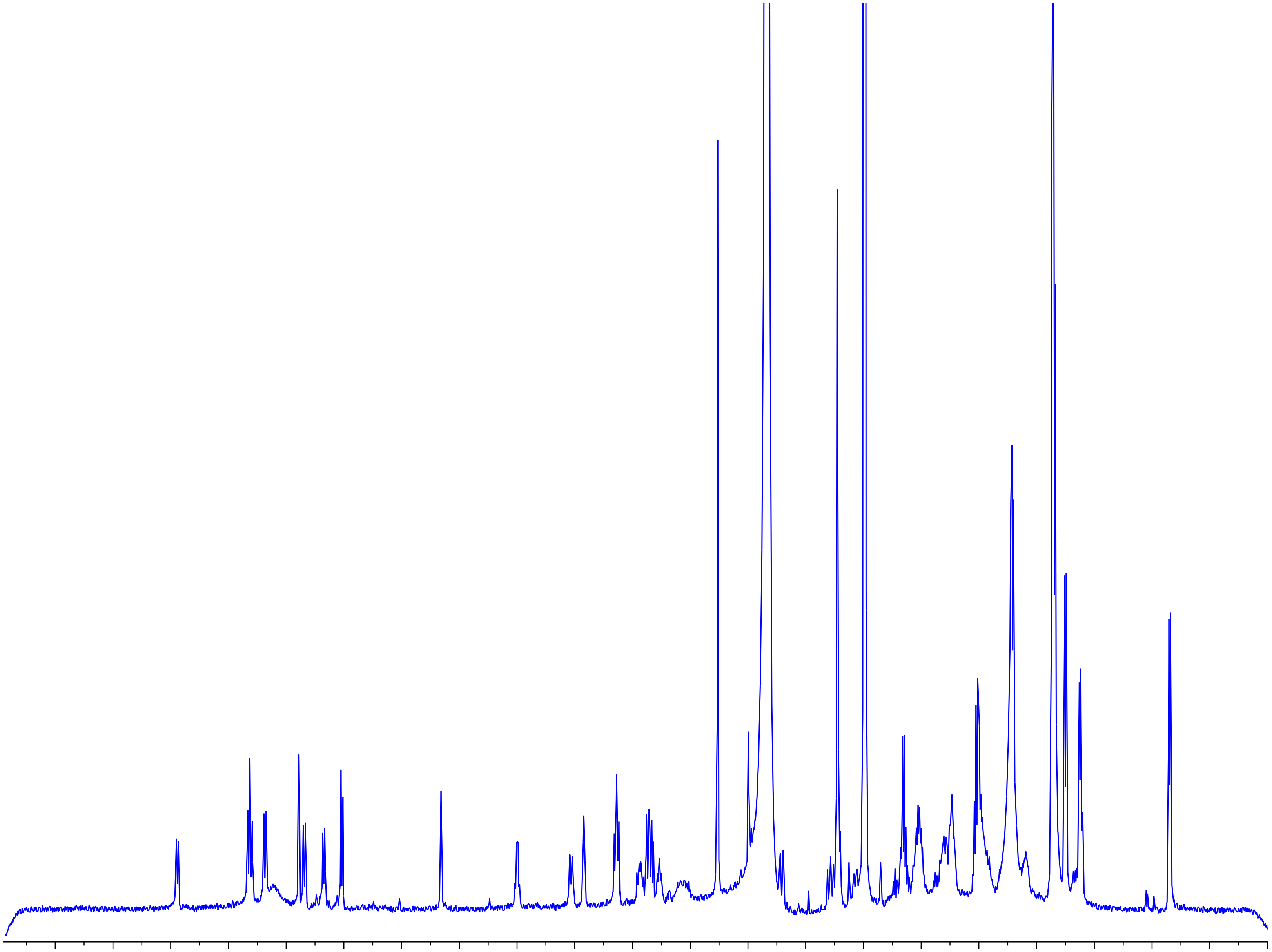

Comparison of ^{1}H NMR spectra of kempopeptins B (**2**) and C (**3**) in DMSO-d_6 (600 MHz)

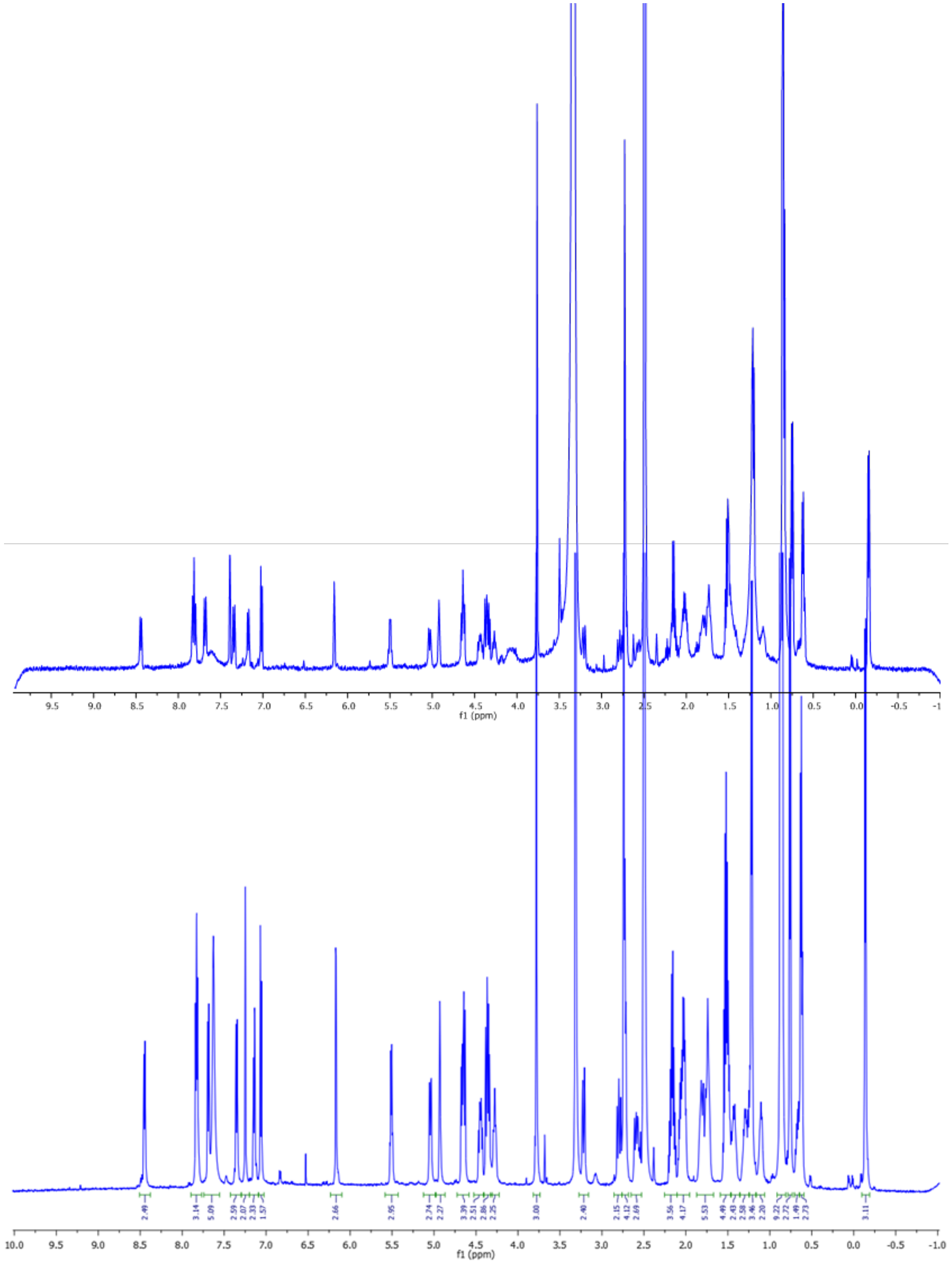

kempopeptins B (**2**)

kempopeptins C (**3**)

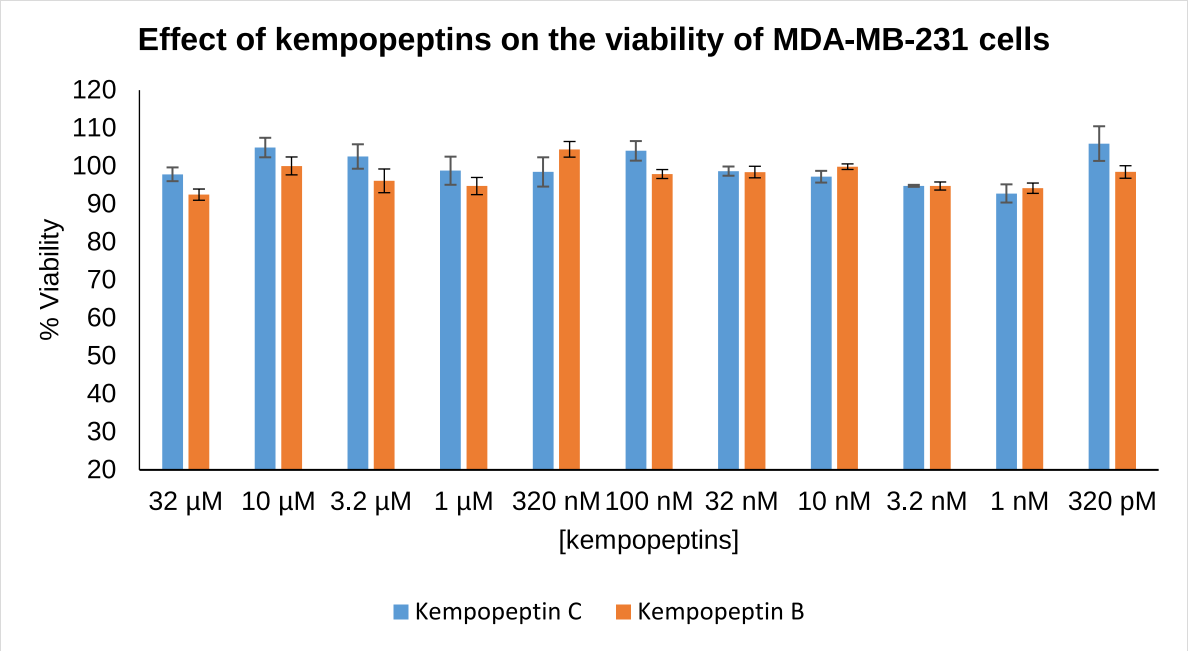

Figure S1. Effect of kempopeptins B and C (**2**) and (**3**) on the viability of MDA-MB-231 breast cancer cells.

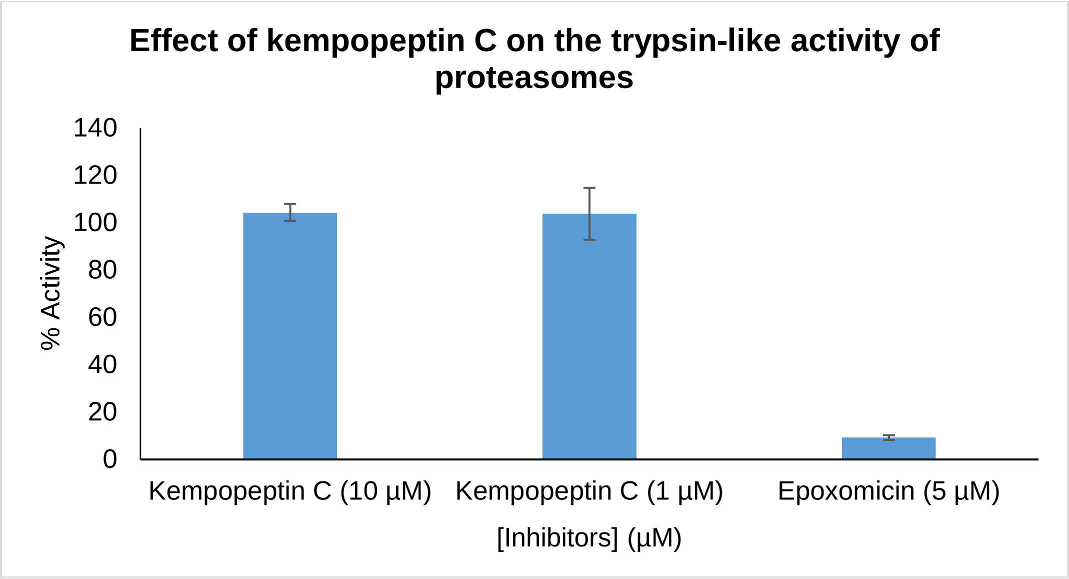

Figure S2. Effect of kempopeptin C (**3**) on the proteasome activity *in vitro* assessed using proteasome Glo trypsin-like activity assay (Promega).
